# Securitization as a means to pay for cell and gene therapies for orphan diseases: a simulation study

**DOI:** 10.1038/s41434-026-00604-6

**Published:** 2026-03-11

**Authors:** John M. Lu, Avi J. Cherla, Alexander W. Carter, Elias A. Mossialos

**Affiliations:** LSE Health, Department of Health Policy, London School of Economics, London, UK

**Keywords:** Diseases, Drug delivery

## Abstract

Cell and gene therapies may provide life-extending treatments for patients. However, paying for these therapies using a single upfront payment will be challenging because of uncertainty about long-term clinical effectiveness and affordability. Developers, recognizing the challenges of paying for these therapies, have offered payers 5-year outcomes-based installment plans. The short length of these plans, however, does little to address uncertainties about the cost-effectiveness of paying for these therapies. Instead, we propose to offer 30-year performance-based annuities that shift payments to match the expected accrual of clinical benefits more closely. Using securitization techniques combined with long-term performance-based annuities, we demonstrate that in the case of the gene therapy Zolgensma, this mechanism is effective at mitigating concerns over value and affordability for payers. In summary, our proposal for financing cell and gene therapies creates a viable incentive for developers, while also balancing long-term effectiveness and budget impact concerns from payers and access challenges for patients.

## Introduction

The US Food and Drug Administration estimated that by 2025, as many as 20 cell and gene therapies would be approved each year [1]. For conditions like spinal muscular atrophy and beta-thalassemia, approved cell and gene therapies offer potential long-term incremental health gains, which are orders of magnitude higher than those of biologics and small molecule drugs [2, 3]. However, the high cost of these treatments, which often exceeds $1 M per treatment, poses challenges for health systems to finance, price, and reimburse these treatments. Despite justifiable concerns about affordability, the financial sustainability of these durable therapies for the health system at large is achievable. For instance, studies project that durable therapies will cost the US health system around $20 billion per year by 2030, which is less than 10% of the current US pharmaceutical budget [4, 5]. However, major concerns over access and affordability for individual patients persist because current payment approaches misalign incentives for patients, payers, and drug developers [6]. There exists a major need to reform the specific payment models for these new therapeutic modalities to expand patient access, ensure payer affordability (budget impact and long-term effectiveness), and appropriately incentivize therapeutic breakthroughs for developers.

**Fig. 1:**
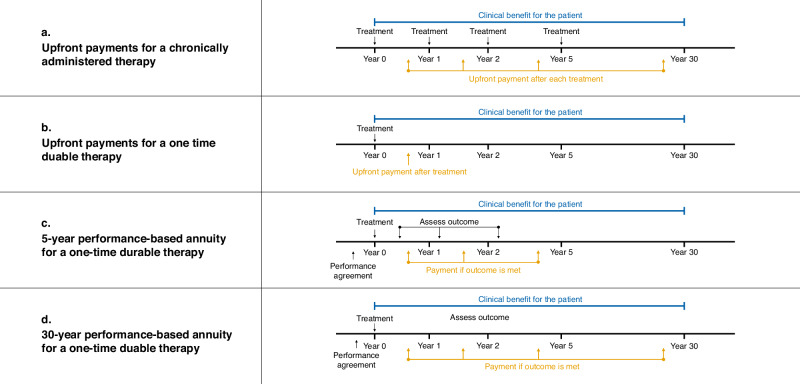
Comparison of the structure of various payment models. **a** Paying for traditional chronically administered therapies using upfront payments results in the regular incurring of costs that roughly matches the continuous accrual of clinical benefits. **b** Paying for one-time durable therapies using a single upfront payment results in a large one-time cost with no guarantee of future clinical benefit. **c** 5-year performance-based annuities spread the cost of therapy over 5 outcomes-based installments but does not guarantee clinical benefit after the initial 5 years. **d** 30-year performance-based annuities spread the cost of therapy over 30 outcomes-based installments. The regular incurring of costs roughly matches the continuous accrual of clinical benefits, as in (**a**).

From the health financing perspective, durable therapies primarily differ from traditional chronic therapies (Fig. 1a) because costs for durable therapies are incurred upfront while benefits are maintained and accrue over many years (Fig. 1b). Since upfront payments do not link the paid price to clinical outcomes, they propagate clinical uncertainties into cost-effectiveness uncertainties. Payers will therefore overpay if the therapies provide lower than expected effectiveness, and drug developers will lack the incentive to produce true therapeutic breakthroughs. Clinical uncertainties arise because these therapies promise decades—or even a lifetime—of clinical benefit based on just a few years of data from small and uncontrolled clinical trials [2]. The combination of large upfront payments with significant clinical uncertainty contrasts with chronic therapies, where the amounts spent are orders of magnitude smaller. In markets like the US, where patients frequently switch insurers, upfront payments result in the original insurer paying the entire cost of therapy [7]. Payers are then disincentivized from covering these therapies, which, while cheaper for health systems over the long-term, are costlier for individual payers in the short-term.

To alleviate some clinical and economic uncertainty, manufacturers have agreed to contracts in which payment is contingent upon continued effectiveness, termed performance-based annuities (PBAs). In theory, PBAs shift the incurring of costs to match the accrual of benefits. In reality, the warranty duration of current PBAs is too short to minimize payers’ overpayment risk [8]. (Fig. 1c). To mitigate the overpayment risk for payers, PBAs should ideally guarantee clinical outcomes for at least the mean duration of the anticipated health gains (Fig. 1d). Long-term PBAs will be challenging to implement, though, because they greatly decrease the size of each payment, thereby lowering the revenue per sale in a calendar year.

In this study, we propose the use of financial securitization, which is the pooling and re-packaging of debt, to improve the feasibility of implementing long-term PBAs. We then use a Monte Carlo simulation to examine the impact of using financial securitization with long-term PBAs on the affordability and value of cell and gene therapies for payers and manufacturers.

## Materials and methods

### Conceptual underpinning: use of securitization to finance performance-based contracts

**Fig. 2:**
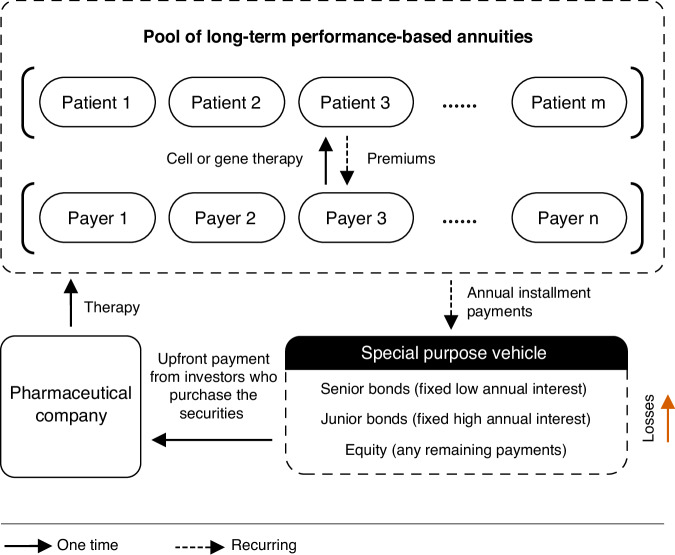
Proposed structure of securitizing 30-year PBAs using cure-backed securities. Before the investment period, payers purchase the drug for their covered patients using long-term performance-based annuities (PBAs). During the one-time investment period, investors purchase the senior and junior bonds, which are used to partially pay the drug company upfront for the durable therapy. During each repayment period, payers pay the annual PBA payments for each patient contingent on continued patient survival to investors instead of the drug company. These payments are distributed to pay senior bondholders first before paying junior bondholders. The pharmaceutical company, which owns the equity securities, receives any leftover money from the annual installment payments.

To improve the design of long-term PBAs, they must increase, rather than decrease, the revenue that companies receive per sale of therapy in the year of sale without increasing the amount paid on average. Our proposal to achieve these goals is for pharmaceutical companies to first sell the therapy on long-term PBA contracts and then to sell the rights to collect annual payments from the PBAs to investors for upfront payment (Fig. 2). Under such an arrangement, pharmaceutical companies will benefit from receiving most of the expected future revenue for the durable therapy upfront, and payers benefit from sustainable financing through long-term PBAs. This would reduce affordability concerns, mitigate cost-effectiveness uncertainties, and eliminate the coverage disincentive. Our proposed implementation of long-term PBAs aligns incentives for pharmaceutical companies, healthcare payers, and investors. An assessment of various payment models and the proposed combination of securitization and long-term PBA is summarized in Table 1.

**Table 1:** Impact of various payment models on payers, pharmaceutical companies, and investors.

Payment model	Stakeholder					
	Payer		Pharma		Investor	
**Upfront payment**	Major challenges: affordability issues, high uncertainty over effectiveness and cost-effectiveness, and coverage disincentives	+	No consequences if therapy has lower than expected effectiveness	+ +++	n/a	
**Short-term PBA**	Helps to address affordability issues, marginally reduces cost-effectiveness uncertainty, but does not address uncertainty over long-term effectiveness. Can address the coverage disincentive	+ +	Minimal consequences if therapy has lower than expected effectiveness past 5 years. Minimally delayed revenue	+ ++		
**Long-term PBA**	Fully addresses affordability issues, fully reduces uncertainty for effectiveness, fully reduces cost-effectiveness uncertainty, and can eliminate the coverage disincentive	+ +++	Full consequences if therapy has lower than expected effectiveness. Extensively delayed revenue	+		
**Long-term PBA + cure-backed security**	Fully addresses affordability, effectiveness, and cost-effectiveness uncertainty. Can eliminate coverage disincentive	+ +++	Moderate consequences if therapy has lower than expected effectiveness. Frontloaded revenue	+ +	+ +++	Offers competitive returns and diversified risk

Crosses denote the relative attractiveness of each payment model for each stakeholder: + = marginally favourable; ++ = moderately favourable; +++ = favourable; ++++ = highly favourable; n/a = not applicable. Assessments reflect the balance of financial risk, revenue timing, and outcome uncertainty under each model.

For PBAs to be more than just a theoretically attractive investment, PBAs must also offer a favorable risk-return profile to investors. To reduce the risk of investing in PBAs—and the premium that investors need to be paid to assume the risk—we propose [1] that the returns of multiple PBAs be pooled to reduce the variability of PBA returns and [2] that investors’ investments be protected from loss if lower-than-expected effectiveness leads to a shorter duration of PBA payments.

First, to pool PBA returns, we propose the use of the common financial engineering technique, securitization, to create cure-backed securities (CBSs), which are modeled on asset-backed securities. Securitization pools different types of debt (or illiquid assets) and sells their revenue-generating potential to investors as securities. Here, the underlying asset is the right to collect annual payments from long-term PBAs. Investors have the choice of purchasing the underlying debt (bonds) or equity (stocks) of the newly generated security. We propose that rather than purchasing individual PBAs, investors can purchase CBSs and receive a portion of the cash payments made to the PBA pool, with the owners of the debt securities (bonds) first receiving fixed annual amortized payments and the owners of the equity securities (stocks) then receiving any leftover cash.

Second, since there is a large uncertainty in the durability of these therapies’ effectiveness, we model a scenario in which pharmaceutical companies will retain the riskier equity securities. If the underlying PBAs pay less than expected due to lower than expected effectiveness, the equity securities held by the pharmaceutical company lose money first and protect the bonds owned by investors. If the therapy is more effective than expected, then the pharmaceutical companies will be rewarded, thereby incentivizing therapeutic breakthroughs. These techniques have previously been proposed to reduce the borrowing costs of personal healthcare loans for curative therapies [9].

### Simulating cure-backed securities

To predict the feasibility of the proposed CBSs, we simulated hypothetical CBSs of 30-year PBAs for Novartis’s (Basel, Switzerland) Zolgensma and assessed the implications for the pharmaceutical company, payers, and investors. We simulated the feasibility of our model using Zolgensma because it was one of the first gene therapies approved in the US and had sufficient clinical data available. Payers currently pay for Zolgensma using 5 annual payments of $425,000, with payments contingent on continued patient survival. After adjusting for inflation and expected patient efficacy calculated from a clinical model previously developed by ICER [10]. payers are therefore currently expected to pay $1.8 M current year dollars (CY$) for Zolgensma. In our simulations, for each underlying 30-year PBA, payers are assumed to make annual payments of $130,000 for up to 30 years, as long as the associated patient is alive. After adjusting for inflation and expected clinical efficacy, this 30-year PBA would likewise result in payers paying an average of CY$1.8 M per patient treated with Zolgensma (Fig. S7). We also perform our simulations using the price recommended by ICER of $900,000 [10]. Five hundred PBAs were simulated because 500 infants are expected to be born each year with SMA in the US [11, 12].

### Statistical analysis

We estimated the cash flow implications for the hypothetical CBSs using 1000 Monte Carlo simulations that rely on repeated random sampling from probabilistic distributions to obtain numerical results. In each Monte Carlo simulation, we first simulated the survival times for 500 patients treated with Zolgensma, with the survival times simulated under three effectiveness assumptions: base-case, probabilistic sensitivity analysis (PSA) of the base-case, and pessimistic, using a clinical model previously developed by ICER [10]. (Fig. S5). We then calculated for each repayment period *t*, the amount of incoming payments from the pool of PBAs and the amount of outgoing CBS payments to investors. The amount of incoming PBA payments was calculated from the number of PBA-associated patients still alive at time *t*. The amount of outgoing CBS payments was calculated by distributing the incoming PBA payments to investors. The clinical and financial models are described in detail in the supplemental material. We then averaged the financial performance of the CBSs across the 1000 Monte Carlo simulations.

In our simulations, we set the repayment order such that if the underlying PBAs pay less than expected due to lower than expected effectiveness, senior bondholders are repaid first, followed by junior bondholders. The equity tranche is owned by the company and receives the remaining money after the debt tranches are paid. To compensate for the assumed risk, senior and junior bondholders receive annual amortized coupons, yielding 3.61% and 4.01% respectively. These rates were set to be 0.50% and 0.90% above the average yields of US Treasury Bills in 2018. The equity tranche reaps extra payments if the therapy has higher effectiveness than expected, but fewer payments otherwise. The senior debt, junior debt, and equity tranches were assumed to comprise 40%, 10%, and 50% of the issued CBSs.

The clinical/financial modeling, Monte Carlo simulations, and statistical analyses were performed using Microsoft Excel.

## Results

The Monte Carlo simulation results indicate that the hypothetical CBSs for Zolgensma can offer favorable risk-return profiles for the senior and junior bondholders. Under all three scenarios, the bonds, comprising 50% of the CBSs, have a low probability of default (<0.1%) and low expected losses (<0.1 bps), making them attractive to investors (Figs. S9, 10). In contrast, the equity securities are unattractive because their median internal rate of return is almost always lower than the yield on US Treasury Bills of the same maturity, which are considered risk-free investments. The equity securities (comprising the other 50% of CBSs) will therefore need to be either held by the manufacturer (Novartis) or sold at a discount to create a higher rate of return. If the equity securities are held by the manufacturer, then the manufacturer will be incentivized to produce therapeutic breakthroughs since the payout from the equity securities is greatest if the therapeutic has greater than expected clinical effectiveness.

**Fig. 3:**
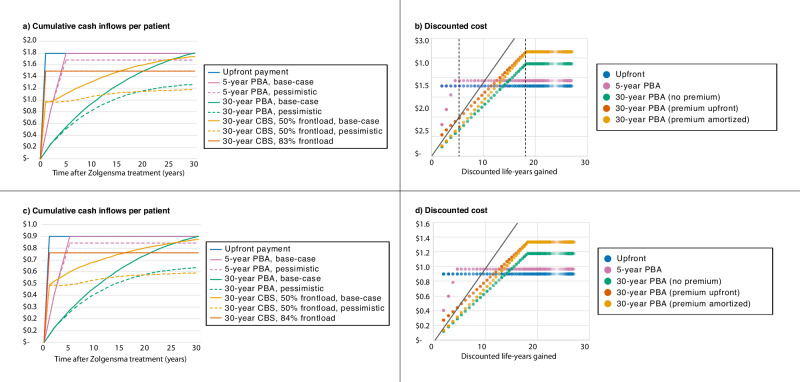
Impact of CBS on pharmaceutical companies and payers. **a** The expected net present value of cumulative cash inflows to Novartis per single sale of Zolgensma from various financing options under base-case (solid line) and pessimistic (dotted line) effectiveness assumptions. The average cumulative amount paid by payers per patient is equal to the cash inflows to Novartis for the upfront payment, 5-year PBA, and 30-year PBA cases. In the 30-year CBS case, payers still pay the same amount they would have in the 30-year PBA case, but Novartis receives its money sooner from the sale of CBSs to investors. **b** Cost-effectiveness plane for Zolgensma from the perspective of payers under different payment plans. The dotted lines illustrate the amount a payer would pay if a patient were to live for either 6 or 30 years after treatment. Intensity of color indicates the relative numbers of patients with a given combination of cost and clinical benefit. **c** As in (**a**), assuming a treatment cost of $900k instead of $1.8 m. **d** As in (**b**), assuming a treatment cost of $900k instead of $1.8 m.

Potential investor interest in CBSs implies that Novartis can use CBSs to partially frontload payments from the hypothetical 30-year PBAs, making the 30-year PBAs more palatable to pharmaceutical companies to implement. The amount frontloaded under the simulated CBS structure can vary from 50% of the PBAs’ expected net present value (ENPV) if Novartis sells the bonds but holds the equity securities (which incurs a 2.85% fee from paying the bondholders interest rates above the discount rate), to a maximum of roughly 83% of the ENPV, achieved if Novartis sells the equity securities at a 33.90% discount to produce an attractive 7.00% annualized expected return for investors (though lower than the 12.5% proposed by Montazerhodjat et al. [9]). (Fig. 3a). Because frontloading is advantageous, CBSs increase the attractiveness of 30-year PBAs to Novartis. The relative preferability of 30-year PBAs implemented with CBSs to existing standalone 5-year PBAs, however, is less certain. On one hand, the frontloaded CBS-coupled 30-year PBAs will allow Novartis to receive over twice the revenue per sale of Zolgensma in the year of sale, as it will otherwise receive through its existing 5-year PBA. On the other hand, the remaining revenue will only be received over another 29 years, instead of 4 years. This inconvenience of delay in payments may be offset by the potential reward for pharmaceutical companies if the therapy has better-than-expected efficacy. For instance, if a treated patient lives a full 30 years after treatment, the payer will pay CY$2,358,350 under a 30-year PBA (30 payments of $130,000 = $3,900,000 before inflation), CY$1,932,560 under a 5-year PBA (5 payments of $425,000 = $2,125,000 before inflation), or CY$1,800,000 in an upfront payment.

Although payers pay more if the therapy has better-than-expected efficacy, 30-year PBA payment plans are highly beneficial for payers because in single-payer systems, long-term PBAs mitigate the significant risk of overpaying if the therapy has worse-than-expected efficacy. For instance, if a hypothetical patient were to only live for six years after treatment instead of the anticipated 30 years, then the payer would pay CY$698,440 under a 30-year PBA (6 payments of $130,000 = $780,000 before inflation), CY$1,932,560 under a 5-year PBA (5 payments of $425,000 = $2,125,000 before inflation), or CY$1,800,000 in an upfront payment. Under more realistic pessimistic effectiveness assumptions, for instance, payers using 30-year PBAs would only pay on average $1.27 M per patient requiring Zolgensma, in contrast to $1.68 M using 5-year PBAs or $1.80 M using upfront payments (Fig. 3a).

The risk mitigation offered by long-term PBAs reduces cost-effectiveness uncertainties due to worse-than-expected efficacy, such that almost no combinations of cost and clinical benefit for the 30-year PBAs are cost-ineffective (Fig. 3b). This result holds even if Novartis were to pass the 17% transaction cost from the 83% frontload onto payers (Fig. 3b), and if Zolgensma’s price were $900,000 (Fig. 3c, d). For individual payers in multi-payer systems (such as the US) in which patients frequently switch insurers and insurers assume the remaining payment obligations of new policyholders, the 30-year PBAs will almost always lower costs for a given insurer, regardless of actual effectiveness, compared to 5-year PBAs and upfront payments. Even if the payer were to pay more for better-than-expected efficacy, the payer would be paying for a guaranteed clinical outcome rather than a promised benefit that may not hold true. As a result, long-term PBAs ensure cost-effectiveness regardless of clinical efficacy by protecting payers against worse-than-expected efficacy and rewarding pharmaceutical companies in the event of better-than-expected efficacy.

## Discussion

In anticipation of the 90,000 patients expected to receive cell and gene therapies over the next decade in the USA alone [4]. New financing options are desperately needed to resolve the challenge between short-term payment contracts and long-term durability concerns. This issue is crucial to the development of the durable therapy market, promoting effective competition by developers and mitigating access issues that result from poorly functioning markets. In our proposed model, we use securitization combined with PBAs to finance the purchase of cell and gene therapies. We simulate the financial performance and feasibility of our model for the case of Zolgensma, and demonstrate that it frontloads revenue for the developer, lowers overpayment risk for payers, and provides a worthwhile opportunity for investors. Our model can be readily applied to finance the purchase of other cell and gene therapies for orphan diseases, for which uncertainties create inefficiencies (as explained in Fig. 1). Beyond making long-term PBAs more commercially attractive to manufacturers by front-loading revenues, securitization may also enhance affordability for payers. By pooling risks and applying tranching, securitization can increase the effective net present value of PBA cash flows above the single-patient baseline, thereby lowering the cost of capital. If structured with the involvement of powerful public payers such as CMS or other national purchasers, this could enable reductions in annual installment payments and improve patient access. The analogy is to the emergence of mortgage-backed securities, which spread long-term risks across capital markets and allowed the development of affordable 30-year fixed-rate mortgages. Similarly, a publicly sponsored CBS mechanism could reduce installment costs for life-saving therapies, aligning the interests of patients, payers, and developers.

### Limitations and further research

Our simulations, however, have limitations. The results of the financial simulations are based on assumptions that were made to overcome the lack of long-term follow-up data for Zolgensma (which is currently being evaluated in several phase 3 trials). In addition to prospective randomized trials, the financialization of outcomes may incentivize the collection of real-world evidence (RWE) of clinical effectiveness, given the financial interests involved in measuring the long-term performance of the associated therapies. A spillover of the proposed cure-backed security (CBS) model, the incentive to use RWE would address fundamental uncertainties and risks associated with gene and cell therapies by accelerating feedback to all stakeholders. For this to happen, governance mechanisms that support the public interest will also need to evolve. The financial model also does not factor in the costs of monitoring individual patient outcomes for the PBAs or operating the special-purpose vehicle. Although likely to be small (<3% for PBA monitoring costs (author’s calculations, see supplemental material) and <0.5% for management fees), these costs do need to be paid and can be shared by all stakeholders. Our simulation also does not model the risk of bankruptcy for healthcare payers.

Conceptually, despite their promise, the securitization of long-term PBAs is not a universal solution to the policy problem of upfront payments. If used as a payment model for non-orphan durable therapies, long-term PBAs will prevent payers from realizing savings for treated patients that result from the market entry of competitor or generic therapies, which is likely to occur because of the large potential market size for non-orphan diseases. However, for most (ultra-)orphan diseases with relatively low incidence, only one gene therapy will likely ever be developed due to the limited number of untreated patients to support clinical trials for a second gene therapy. Long-term PBAs, therefore, are primarily useful as a payment model for genetically driven orphan diseases, for which durable cell and gene therapies are likely to emerge. While the patient populations for individual orphan diseases are comparably small, the cumulative number of patients affected by the thousands of single-gene disorders is large, necessitating this proposed financing mechanism. Further, this form of financing facilitates managing the inevitable shift from competition at the disease level, rather than the product level.

Long-term PBAs primarily address the discontinuity in accrued health gains and the upfront payment dilemma. Neither standalone nor CBS-coupled long-term PBAs directly addresses the excessively high prices of many durable therapies. We have chosen to sidestep an extensive discussion on the prices of durable therapies. Our use of Novartis’s list price for Zolgensma in our simulations, therefore, is only meant for illustrative purposes. Our proposed CBS-coupled long-term PBA financing solution should be implemented alongside standard price-control strategies and value assessment methods. The latter is a highly active area of research and policy discussion, which raises questions about how performance is measured given stakeholder perspectives [13]. Based on the current debate, in the future, value assessment of orphan therapies might extend beyond QALYs and (net) costs by, for example, integrating disease-level equity impacts into valuations. We would expect related developments in RWE and value assessment to inform the performance-based payment associated with a CBS.

Adaptations to the current health policy environment are already underway for cell and gene therapies. Currently, state-run Medicaid programs receive the lowest drug prices under the Medicaid best price law. While this allows Medicaid to effectively negotiate lower drug prices, outcomes-based agreements (such as PBAs) that rebate payment if the treatment is not effective have been prohibited. If a patient covered by Medicaid were to relapse on the potential treatment, the rebated price (zero) would become the de facto price covered by Medicaid. Recently, the US Center for Medicare and Medicaid Services announced that it was adapting its policy to account for the influx of highly expensive, potentially curative cell and gene therapies [14]. This new change would reflect a blend of prices and include alterations to the best price and average manufacturer price outside of 3 years (the time currently allowed for manufacturers to submit price changes to Medicaid), paving the way for exploring new value-based payment options.

## Conclusion

Paying for cell and gene therapies using upfront payments has proved challenging, with risks based on financial sustainability/affordability, high uncertainty about cost-effectiveness, and concerns about access [15]. Current policy solutions are inadequate for dealing with these challenges: the standalone short-term PBAs piloted by Novartis and other pharmaceutical companies minimally mitigate uncertainties, and the previously proposed standalone long-term PBAs—while highly attractive to payers—are unfavorable because they extensively delay receipt of revenue.

We instead propose and empirically test an alternative policy solution—cure-backed securities (CBSs)—to be implemented in conjunction with long-term PBAs to address the problem of upfront payments. Through simulations using Novartis’s Zolgensma, we demonstrate that the securitization of long-term PBAs using CBSs preserves the advantages of long-term PBAs for payers, mitigates the challenges that pharmaceutical companies face in offering long-term PBAs, and provides investors with competitive and diversified returns. Without this, the durable cell and gene therapy market will struggle to mature, increasing the potential for long-term health system inefficiencies that stem from insufficient policy innovation to support technological breakthroughs.

In summary, securitization can act as a viable alternative policy solution to the challenge of paying for durable orphan therapies using upfront payments. With hundreds of gene therapies in human clinical trials and thousands of single-gene disorders amenable to gene therapy [16, 17]. Now is the time to explore the financial innovations needed to enable payment for, and access to, breakthrough therapies. In addition to improving the feasibility of long-term PBAs for manufacturers, securitization - particularly if supported by national payers- could lower installment costs and directly strengthen affordability and access for patients.

## Supplementary information

Supplementary Information

## Data Availability

Analyses are based on publicly available data.
