# Supplementary material for: Securitization as a means to pay for cell and gene therapies for orphan diseases: a simulation study
Source: Gene Ther. 2026 Mar 11;33(2):138–43. doi: 10.1038/s41434-026-00604-6 (PMC13056534; doi:10.1038/s41434-026-00604-6)
Supplement: Supplementary file 1 — Supplementary Information [file 41434_2026_604_MOESM1_ESM.docx]

# Supplementary Information

[Improving the risk-return profile of PBAs through securitization and credit-enhancement 2](#_Toc75525976)

[Clinical model of type 1 spinal muscular atrophy 4](#_Toc75525977)

[Clinical model data inputs and assumptions 5](#_Toc75525978)

[Clinical model outputs and sensitivity/scenario analyses 6](#_Toc75525979)

[Financial model of cure-backed securities 7](#_Toc75525980)

[Financial model data inputs and assumptions 7](#_Toc75525981)

[Financial model data outputs and sensitivity/scenario analyses 9](#_Toc75525982)

[Clinical model results 9](#_Toc75525983)

[Cure-backed security performance and cost-effectiveness analysis 11](#_Toc75525984)

[Criticisms of performance-based annuities 15](#_Toc75525985)

[Supplementary references 23](#_Toc75525986)

Improving the risk-return profile of PBAs through securitization and credit-enhancement

For PBAs to be more than just a theoretically attractive investment, PBAs must also offer a favorable risk-return profile to investors. One strategy for improving the risk-return profile is the pooling together of multiple PBAs, which reduces the variability of returns by reducing the variability in clinical outcomes for the underlying patient population. Figure S1 shows how increasing pool size decreases the variability of returns for hypothetical 30-year PBAs for Novartis’s Zolgensma. The pool of PBAs can then be sold to investors in portions, with each portion being a cure-backed security (CBS) that entitles the owner to receive cash proceeds from the PBA pool. Importantly, a special purpose vehicle (SPV) must pool the PBAs because the SPV will legally isolate the PBAs from the original pharmaceutical company, thereby protecting the PBAs in the event of pharmaceutical company bankruptcy. The SPV’s legal protection is crucial for improving the PBAs’ risk-return profile. This process of transforming illiquid assets that generate regular flows of cash payments into highly liquid asset is termed securitization.


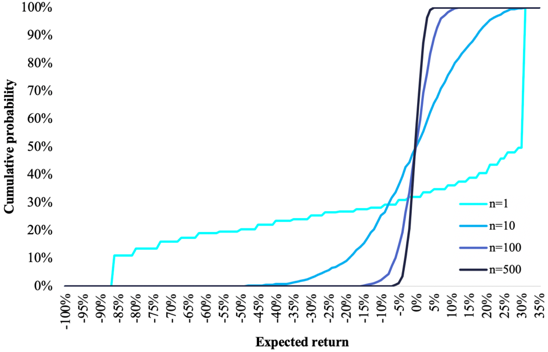


Figure S1: The impact of the size of a pool of PBAs on the variability in returns of the pool.

Investor risk from investing in PBAs can be further reduced using the financial engineering technique of credit enhancement. One common method of credit enhancement is the subordination of certain tranches of notes to others so that if the underlying PBAs pay less than expected, the more junior tranches lose money first and protect the more senior tranches. The proposed CBS uses a subordinated structure, issuing three tranches of notes (in decreasing order of seniority): senior notes, junior notes, and equity notes. The notes in the senior and junior tranches are debt securities, which provide regular amortized payments to investors at preset fixed rates of return. The equity tranche receives any remaining money after the debt tranches are paid. The proposed CBS also uses reserve accounts, another form of internal credit enhancement, to help ensure timely payments to the debt tranches. The final form of internal credit enhancement used in the proposed CBS are the interest coverage test and the principal coverage (overcollateralization) test (see Figure S6 for details). These coverage tests are designed to ensure that the incoming cash from the PBAs is sufficient to cover the scheduled debt tranche payments. If not, cash is diverted to pay down the principal of the debt tranches to reduce the size of scheduled payments. Figure S2 depicts the impact of these credit enhancement techniques on how cash payments from the underlying PBAs are disbursed to the debt and equity tranches.


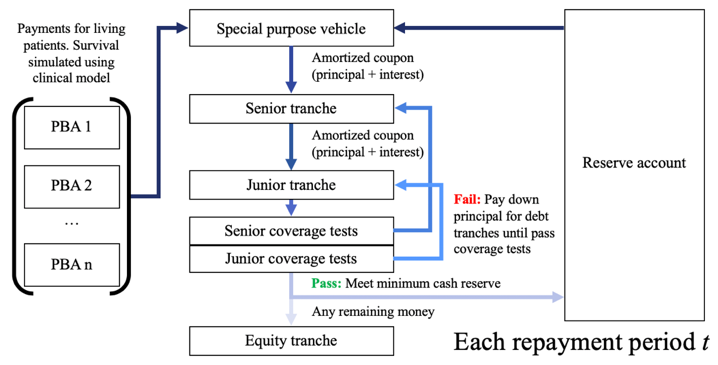


Figure S2: Cash waterflow of proceeds from performance-based annuity payments.

Clinical model of type 1 spinal muscular atrophy

Clinical efficacy is modeled because the financial performance of CBSs constructed from *n* PBAs for Zolgensma is dependent on the clinical outcomes of the *n* associated patients. The clinical efficacy of Zolgensma for patients with type 1 SMA was modeled by reconstructing a model developed previously by the Institute for Clinical and Economic Review (ICER)^1^. ICER’s clinical model has two components: a short-term model based on Zolgensma clinical trial data and a long-term extrapolation model. This paper’s reconstructed model (Figure S3), described here, uses the same structure and underlying assumptions, though with several simplifications. The reconstructed short-term model begins with treatment at birth, even though the average age of Zolgensma administration was 3.4 months (0.9-7.9 months) in clinical trials^2^. Patients then progress to one of four clinical states: *not sitting*, *sitting*, *walking*, and *death* at two time points: ages 1 and 2. Transition is only allowed to the same or improved health state at age 2. This design should not upwardly bias results since the transition probabilities between states are chosen to reflect the observed distribution of health states in clinical trials at age 2, the beginning of the long-term extrapolation model. After age 2, due to a lack of clinical trial data, clinical efficacy is modeled by extrapolating the motor function milestones over the lifetime of each patient.


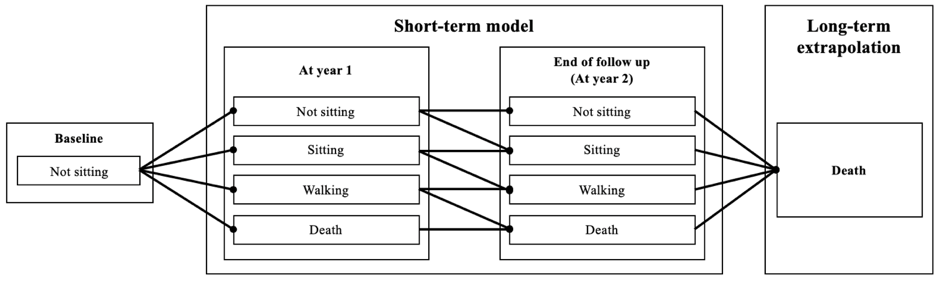


Figure S3: Modified clinical model of type 1 spinal muscular atrophy developed originally by the Institute for Clinical and Economic Review.

Clinical model data inputs and assumptions

Figure S4 presents all base-case clinical model data inputs. Transition probabilities for the short-term model are estimated based on ICER’s description of its model. Probabilities of transition to death in the long-term model are modelled using survival curves that ICER developed from mortality data of patient populations assumed to be similar to the patients in each health state. Figure S5 plots the assumed survival curves.

The reconstructed clinical model, like the ICER model, makes the following key assumptions: (1) all treated patients survive to age 2, reflecting the clinical data from the pivotal 12-patient Zolgensma single-arm trial^2^, (2) patients maintain the motor function milestones achieved at the end of the short-term model for life, (3) treated patients in the *sitting* state have mortality similar to SMA Type II patients, and (4) treated patients in the *walking* state have mortality similar to SMA Type III patients, which is similar to general population mortality. For a full discussion of these assumptions, see ICER’s description of its model^1^.

Figure S4: Data inputs for the clinical model, adapted from ICER’s model

| **Short-term clinical model after treatment with Zolgensma** | | | |
| --- | --- | --- | --- |
|  | **Base case value** | **Range (one-way sensitivity analysis)** | **Distribution (probabilistic sensitivity analysis)** |
| Probability of *death* at year 1 | 0.0% |  |  |
| Probability of *not sitting* at year 1 | 65.0% | 52.0%-78.0% | Beta |
| Probability of *sitting* at year 1 | 30.0% | $=\frac{6}{7}\left( 1-Pr(not sitting y1) \right)$ |  |
| Probability of *walking* at year 1 | 5.0% | $=\frac{1}{7}\left( 1-Pr(not sitting y1) \right)$ |  |
| Probability of *not sitting* at year 2 if *not sitting* at year 1 | 32.0% | 25.6%-38.4% | Beta |
| Probability of *sitting* at year 2 if *not sitting* at year 1 | 68.0% | $=1-Pr\left( not sitting y2 \right\vert not sitting y1)$ |  |
| Probability of *sitting* at year 2 if *sitting* at year 1 | 61.0% | 48.8%-73.2% | Beta |
| Probability of *walking* at year 2 if *sitting* at year 1 | 39.0% | $=1-Pr\left( sitting y2 \right\vert sitting y1)$ |  |
| Probability of *walking* at year 2 if *walking* at year 1 | 100.0% |  |  |
| **Long-term clinical model after treatment with Zolgensma** | | | |
| **Clinical State** | **Assumption** | **Mortality curve source** | **Distribution** |
| Not Sitting | Mortality is assumed to be same as that of patients placed on non-invasive respiratory muscle aid. | Gregoretti et al^3^ (NRA curve) | Exponential (λ=0.0158) |
| Sitting | Mortality is assumed to be same as that of SMA Type II patients. | Zerres et al^4^ | Gompertz  (α=0.0037, β=0.0964) |
| Walking | Mortality is assumed to be same as that of the general population. | US population mortality^5^ |  |


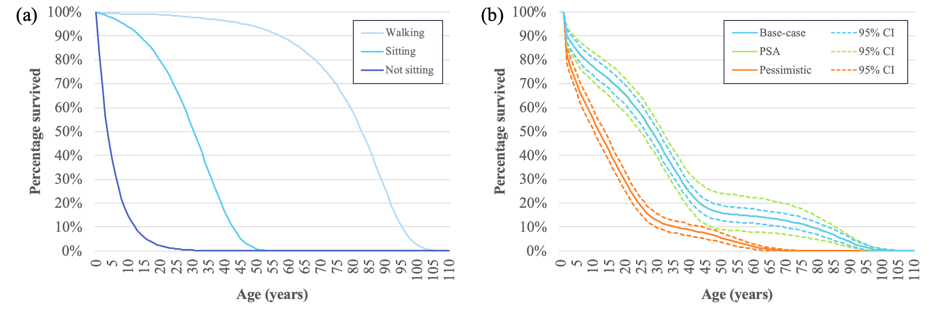


Figure S5: Assumptions used in simulations of CBS financial performance.

(a) Assumed survival curves for SMA Type 1 patients treated with Zolgensma achieving the “walking”, “sitting”, and “not sitting” motor milestones^1^.

**(b)** Simulated net survival curves for a group of 500 treated patients under base-case, probabilistic base-case, and pessimistic efficacy assumptions.

Clinical model outputs and sensitivity/scenario analyses

Modeling of the financial performance of CBSs constructed from *n* PBAs requires modeling of the clinical outcomes of the *n* associated patients. To estimate the distribution of outcomes for a group of *n* patients, the survival times for *n* patients were simulated 1,000 times, with the underlying clinical parameters held constant between simulations. The resulting distribution captures the variation from having a finite population.

To test the sensitivity of the estimated survival distribution to clinical parameter uncertainty, a one-way sensitivity analysis was performed in which clinical parameters were varied ±20%, as per ICER’s simulations. The true uncertainty is likely greater. Only three parameters were varied because the other transition probabilities are derived from these three parameters. A multi-way sensitivity analysis was also performed by simultaneously varying all three parameters to minimize and maximize the mean age of death. A probabilistic sensitivity analysis (PSA) was performed by jointly varying all clinical parameters between each of the 1,000 Monte Carlo simulations to quantify the combined effect of uncertainty of each parameter. Parameter bounds and associated distributions are listed in Figure S4.

A pessimistic scenario analysis was performed, as per ICER’s simulations, to account for the lack of long-term follow up data and the model’s optimistic assumptions. This scenario assumes that (1) the survival times for the *sitting* and *walking* states are halved, implemented using hazard ratios of 5 and 16 respectively, and (2) 30% of patients in the *sitting* state transition to the *not sitting* state at age 2, implemented by setting the probability of *not sitting* at year 2 if *not sitting* at year 1 to 47.4%.

Financial model of cure-backed securities

To assess the financial performance of a hypothetical CBS constructed of *n* pooled PBAs for Zolgensma, a de novo financial model was constructed. The model simulates investors’ returns for the debt and equity tranches of the CBS by simulating for each repayment period *t*, the amount of incoming payments from the pool of PBAs and the amount of outgoing payments to investors, with payments distributed as detailed in Figure S2. The amount of incoming PBA payments per repayment period is calculated based on the simulated survival times for the *n* associated patients.

Financial model data inputs and assumptions

Figure S6 presents the data inputs for the base-case CBS financial model (other simulated CBSs differ only in tranche composition). The simulated CBS receives payments from 500 30-year PBAs for Zolgensma. Each PBA provides 30 annual payments of $130k, with payments contingent on the continued survival of the associated patient. The PBA installment size is set such that the ENPV of the 30-year PBA is equal to the ENPV of the 5-year PBA that Novartis currently offers (Figure S7). The simulated CBS disburses payments to three tranches: a senior debt tranche (40%), a junior debt tranche (10%), and an equity tranche (50%). The senior and junior tranches receive annually compounding interest rates of 3.61% and 4.01% respectively, which are 50 and 90 basis points (1 bp = 0.01%) above market rates. Market rates for a given maturity are set as the average yields of US Treasury Bills with the same maturity in 2018^6^.

The financial model does not factor in management fees for the CBS and monitoring costs for the patients. The model also makes the following assumptions: (1) all incoming and outgoing payments are received and disbursed on the same day, (2) payers stop making PBA payments only if the associated patient dies, and (3) the CBS receives PBA payments regardless of whether the underlying patient switches insurers. The validity of these assumptions is discussed in the limitations section of the discussion in the main text.

Figure S6: Data inputs for the base-case financial model

| **Simulated performance-based annuity for Zolgensma** | | | |
| --- | --- | --- | --- |
| Expected net present value | $1,795,626 |  |  |
| Term length | 30 years |  |  |
| Interest rate | 5.97% |  |  |
| Discount rate | Market rate + 50 bps (3.61%) |  |  |
| Payment stops when | Patient death |  |  |
| Annual payment | $130,010 |  |  |
| **Cure-backed security for Zolgensma** | | | |
|  | Senior debt tranche | Junior debt tranche | Equity tranche |
| Composition | 40% | 10% | 50% |
| Total sale price | $359.1m | $89.8m | $448.9m |
| Interest rate | Market rate + 50 bps (3.61%) | Market rate + 90 bps (4.01%) | n/a |
| Interest coverage test ratio | $=1+\frac{\% equity tranche}{\% senior tranche}$  (1.20) | $=1+\frac{\% equity tranche}{\% debt tranches}$  (1.18) | n/a |
| Overcollateralization test ratio |  |  | n/a |
| Number of underlying contracts | 500 |  |  |
| Market rate, 1-year maturity | 2.33% |  |  |
| Market rate, 5-year maturity | 2.75% |  |  |
| Market rate, 30-year maturity | 3.11% |  |  |

Figure S7: Impact of term length on hypothetical performance-based annuities for Zolgensma

| **Term length** | **Upfront (hypothetical)** | **5-years (existing)** | **30-years (hypothetical)** |
| --- | --- | --- | --- |
| Cost of therapy^*^ | $1.796m | | |
| Annual payment, assuming efficacy | $1.796m | $425k | $130k |
| Interest rate^*^ | n/a | 5.89% | 5.97% |
| Discount rate^*^ |  | 3.25% | 3.61% |
| Maximum total payments | $1.796m | $2.125m  ($1.796m principal + $329k interest) | $3.900m  ($1.796m principal + $2.105m interest) |
| Present value of maximum total payments^†^ | $1.796m | $1.933m | $2.358m |
| Expected net present value^‡^ | $1.796m | $1.796m | $1.796m |
| ^*^The cost of therapy is set at the expected net present value (ENPV)^‡^ of Novartis’s current 5-year PBA, assuming that payment is contingent on continued patient survival and not on another clinical endpoint. The discount rate used to calculate the ENPV is set at 50 basis points (1 bp = 0.01%) above the 2018 average yield on US Treasury notes of the same maturity (see Section 4.4.1). The interest rate of the 30-year PBA was set such that the ENPV of the hypothetical 30-year PBA equaled the ENPV of the existing 5-year PBA.  ^†^Present value accounts for the impact of inflation.  ^‡^Expected net present value accounts for inflation and expected efficacy. Expected efficacy was calculated based on a clinical model of Zolgensma efficacy developed by the Institute for Clinical and Economic Review. | | | |

Financial model data outputs and sensitivity/scenario analyses

To quantify the financial performance of the simulated CBS, performance metrics were calculated by averaging outputs over 1,000 Monte Carlo simulations, with the underlying clinical parameters held constant between Monte Carlo simulations. The calculated performance metrics include the probability of default/expected loss for the debt tranches and the distribution of internal rates of return for the equity tranche.

To test the sensitivity of these performance metrics to clinical parameter uncertainty, one-way, multi-way, and probabilistic sensitivity analyses and a pessimistic scenario analysis were performed using the same methods as described for the clinical model sensitivity/scenario analyses.

Clinical model results

Figure S5 plots the distribution of clinical outcomes for a group of 500 type 1 SMA patients treated with Zolgensma (blue; Figure S5b), as predicted by a clinical model of Zolgensma efficacy reconstructed from a model previously developed by ICER. Clinical outcomes for a group of 500 patients are simulated because the financial performance of CBSs constructed from 500 PBAs is dependent on the clinical outcomes of the 500 associated patients. There exists variation in the predicted clinical outcomes because the group size is finite. Three main periods of patient death are observed: around ages 2, 32, and 88. These periods correspond to the mean life expectancy of the *not sitting*, *sitting*, and *walking* states. The predicted mean and median ages of death for a population of 500 treated patients are 32.23 (95% CI: 30.00-34.35) and 29.01 (95% CI: 27.00-31.00), which are comparable to the mean age of death of 33.13 predicted by ICER^1^.

The results of the one-way sensitivity analysis on the mean age of death are presented in Figure S8. The greatest decrease in mean age of death (from 32.23 to 28.14 years) occurs when the probability of *not sitting* at age 1 is set at 78%. Under the multi-way sensitivity analysis, the mean age of death falls to 25.89 years. Under the pessimistic scenario (orange; Figure S5b, the mean age of death falls further to 16.18 years. Under the probabilistic sensitivity analysis (green; Figure S5b), the mean and median ages of death are 32.16 (95% CI: 27.19-37.64) and 28.91 (95% CI: 25.00-32.00). The results from the sensitivity/scenario analyses demonstrate that the average age of death is highly sensitive to changes in the clinical model parameters. This sensitivity has implications for the predictability and financial viability of any CBS constructed from such PBAs.


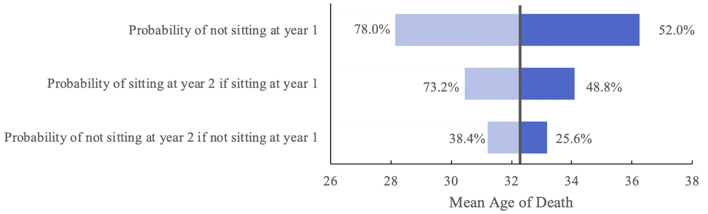


Figure S8: One-way sensitivity analysis of the effect of clinical model parameters on mean age of death.

Thick black line indicates the mean age of death using base case clinical model parameters.

Cure-backed security performance and cost-effectiveness analysis

Figure S9 presents the performance metrics for three hypothetical CBSs (differing in tranche composition) constructed from 500 hypothetical 30-year PBAs for Zolgensma whose payments are contingent upon continued patient survival. For each tranche composition, performance metrics for two models are presented: a deterministic model in which underlying clinical parameters are deterministic and a probabilistic model in which underlying clinical parameters vary according to the distributions used for the clinical model PSA. The deterministic financial model captures the variation from constructing a CBS from a finite number of PBAs. The probabilistic financial model further captures the variation from underlying clinical uncertainty.

These results show that the debt tranches have a non-zero though low probability of default (<1%) and low expected loss (<10 bps). Probability of default and expected loss are highest for the CBS with a 15% equity tranche whose performance is simulated using the probabilistic model. This finding is expected because (1) greater equity tranche size provides greater loss protection for the debt tranches due to subordination, and (2) the probabilistic model unlike the deterministic model captures underlying clinical uncertainties. These results indicate that the debt tranches will likely be attractive to investors.

In contrast, equity tranche performance is poor, providing in nearly all scenarios a median internal rate of return (IRR) less than the yield on US Treasury Bills of the same maturity (the market rate), which are considered risk-free investments. Further, there is a nontrivial probability (>4%) that the IRR for the equity tranche is negative for a CBS with a 15% equity tranche. These results indicate that the equity tranche will likely be highly unappealing to investors and will have to be held by the pharmaceutical company.

The results of the one-way sensitivity analysis, the multi-way sensitivity analysis, and the pessimistic scenario analysis are presented in Figure S10 for the three CBS tranche compositions. The PSA results are presented as the probabilistic model results in Figure S9. For the sensitivity/scenario analyses, probability of default and expected losses for the debt tranches increase as the mean age of patient death decreases. For the multi-way sensitivity analysis, in which mean age of death decreases to 25.89, the probability of default for the debt tranches is high (>30%) for the CBS with a 15% equity tranche, and is low (0%) for the CBSs with 30% and 50% equity tranches. For the pessimistic scenario analysis, in which mean age of death decreases to 16.18, the probability of default for the debt tranches is nearly 100% for the CBSs with a 15% and a 30% equity tranche, and is low (0%) for the CBS with a 50% equity tranche.

Figure S11 presents the incremental cost-effectiveness ratios calculated for upfront, 5-year PBA, and 30-year PBA payment models under base-case and pessimistic efficacy assumptions. These calculations include non-treatment healthcare costs incurred by patients whose lives are extended by Zolgensma but whose survival requires continued and potentially expensive medical support.

Figure S9: Financial performance of the simulated cure-back securities

| Tranche composition | S: 75%, J: 10%, E: 15% | | S: 60%, J: 10%, E: 30% | | S: 40%, J: 10%, E: 50% | |
| --- | --- | --- | --- | --- | --- | --- |
| Model | D | P | D | P | D | P |
| Senior tranche: PD, EL | 0.00%, 0.00bps | 0.40%,  -0.29bps | 0.00%, 0.00bps | 0.00%, 0.00bps | 0.00%, 0.00bps | 0.00%, 0.00bps |
| Junior tranche: PD, EL | 0.00%, 0.00bps | 0.60%,  -6.4bps | 0.00%, 0.00bps | 0.00%, 0.00bps | 0.00%, 0.00bps | 0.00%, 0.00bps |
| Expected IRR (%) | 2.00 | 1.42 | 2.81 | 2.82 | 3.11 | 3.06 |
| Median IRR (%) | 2.04 | 2.08 | 2.79 | 2.85 | 3.13 | 3.06 |
| Standard deviation IRR (%) | 1.15 | 7.00 | 0.58 | 1.06 | 0.36 | 0.65 |
| Pr(complete loss) (%) | 0.00 | 0.00 | 0.00 | 0.00 | 0.00 | 0.00 |
| Pr(IRR < 0) (%) | 4.60 | 18.70 | 0.00 | 0.30 | 0.00 | 0.00 |
| Pr(IRR ≥ 0) (%) | 95.40 | 81.30 | 100.00 | 99.70 | 100.00 | 100.00 |
| Pr(IRR ≥ 2) (%) | 51.40 | 51.10 | 92.40 | 78.80 | 99.90 | 93.90 |
| Pr(IRR ≥ 5) (%) | 0.20 | 8.30 | 0.00 | 1.50 | 0.00 | 0.00 |
| Pr(IRR ≥ 10) (%) | 0.00 | 0.00 | 0.00 | 0.00 | 0.00 | 0.00 |
| S: senior debt tranche, J: junior debt tranche, E: equity tranche, D: deterministic model, P: probabilistic model, PD: probability of default, EL: expected loss, IRR: internal rate of return, Pr(*x*): probability of *x*, bp: basis points | | | | | | |

Figure S10: One-/multi-way sensitivity analysis and scenario analysis of the financial performance of simulated cure-back securities for various CBS tranche compositions:

(a) S: 75%, J: 10%, E:15%, (b) S: 60%, J: 10%, E: 30%, and (c) S: 40%, J: 10%, E: 50%.

| (a) | S: 75%, J: 10%, E: 15% (Deterministic model) | | | | | |
| --- | --- | --- | --- | --- | --- | --- |
| Clinical parameter of interest | Normal | One-/multi-way sensitivity analyses | | | | Pessimistic scenario analysis |
|  |  | Probability of not sitting at year 2 if not sitting at year 1 | Probability of sitting at year 2 if sitting at year 1 | Probability of not sitting at year 1 | Changing of all three clinical parameters |  |
| New parameter value |  | 38.4% | 73.2% | 78.0% |  |  |
| Mean age of death (years) | 32.23 | 31.20 | 30.44 | 28.14 | 25.89 | 16.18 |
| Senior tranche: PD, EL | 0.00%, 0.00bps | 0.00%, 0.00bps | 0.00%, 0.00bps | 0.20%,  -0.02bps | 34.30%,  -24.01bps | 100.00%,  -2043bps |
| Junior tranche: PD, EL | 0.00%, 0.00bps | 0.00%, 0.00bps | 0.00%, 0.00bps | 0.60%,  -2.54bps | 43.30%,  -460bps | 100.00%,  -5764bps |
| Expected IRR (%) | 2.00 | 1.53 | -0.28 | -1.86 | -38.90 | -83.90 |
| Median IRR (%) | 2.04 | 1.57 | -0.12 | -1.09 | -7.83 | -83.90 |
| Standard deviation IRR (%) | 1.15 | 1.19 | 1.59 | 6.68 | 39.16 | 0.00 |
| Pr(complete loss) (%) | 0.00 | 0.00 | 0.10 | 0.00 | 0.50 | 0.00 |
| Pr(IRR < 0) (%) | 4.60 | 9.60 | 52.60 | 76.00 | 99.20 | 100.00 |
| Pr(IRR ≥ 0) (%) | 95.40 | 90.40 | 47.30 | 24.00 | 0.30 | 0.00 |
| Pr(IRR ≥ 2) (%) | 51.40 | 36.60 | 4.30 | 0.80 | 0.00 | 0.00 |
| Pr(IRR ≥ 5) (%) | 0.20 | 0.20 | 0.00 | 0.00 | 0.00 | 0.00 |
| Pr(IRR ≥ 10) (%) | 0.00 | 0.00 | 0.00 | 0.00 | 0.00 | 0.00 |

| (b) | S: 60%, J: 10%, E: 30% (Deterministic model) | | | | | |
| --- | --- | --- | --- | --- | --- | --- |
| Clinical parameter of interest | Normal | One-/multi-way sensitivity analyses | | | | Pessimistic scenario analysis |
|  |  | Probability of not sitting at year 2 if not sitting at year 1 | Probability of sitting at year 2 if sitting at year 1 | Probability of not sitting at year 1 | Changing of all three clinical parameters |  |
| New parameter value |  | 38.4% | 73.2% | 78.0% |  |  |
| Mean age of death (years) | 32.23 | 31.20 | 30.44 | 28.14 | 25.89 | 16.18 |
| Senior tranche: PD, EL | 0.00%, 0.00bps | 0.00%, 0.00bps | 0.00%, 0.00bps | 0.00%, 0.00bps | 0.00%, 0.00bps | 99.90%,  -709bps |
| Junior tranche: PD, EL | 0.00%, 0.00bps | 0.00%, 0.00bps | 0.00%, 0.00bps | 0.00%, 0.00bps | 0.00%, 0.00bps | 100.00%,  -2693bps |
| Expected IRR (%) | 2.81 | 1.77 | 2.59 | 1.40 | -0.05 | -89.19 |
| Median IRR (%) | 2.79 | 1.77 | 2.62 | 1.39 | -0.02 | -89.19 |
| Standard deviation IRR (%) | 0.58 | 0.60 | 0.58 | 0.62 | 0.71 | 0.00 |
| Pr(complete loss) (%) | 0.00 | 0.00 | 0.00 | 0.00 | 0.00 | 0.00 |
| Pr(IRR < 0) (%) | 0.00 | 0.10 | 0.00 | 1.30 | 51.60 | 100.00 |
| Pr(IRR ≥ 0) (%) | 100.00 | 99.90 | 100.00 | 98.70 | 48.40 | 0.00 |
| Pr(IRR ≥ 2) (%) | 92.40 | 35.70 | 83.60 | 17.70 | 0.00 | 0.00 |
| Pr(IRR ≥ 5) (%) | 0.00 | 0.00 | 0.00 | 0.00 | 0.00 | 0.00 |
| Pr(IRR ≥ 10) (%) | 0.00 | 0.00 | 0.00 | 0.00 | 0.00 | 0.00 |

| (c) | S: 40%, J: 10%, E: 50% (Deterministic model) | | | | | |
| --- | --- | --- | --- | --- | --- | --- |
| Clinical parameter of interest | Normal | One-/multi-way sensitivity analyses | | | | Pessimistic scenario analysis |
|  |  | Probability of not sitting at year 2 if not sitting at year 1 | Probability of sitting at year 2 if sitting at year 1 | Probability of not sitting at year 1 | Changing of all three clinical parameters |  |
| New parameter value |  | 38.4% | 73.2% | 78.0% |  |  |
| Mean age of death (years) | 32.23 | 31.20 | 30.44 | 28.14 | 25.89 | 16.18 |
| Senior tranche: PD, EL | 0.00%, 0.00bps | 0.00%, 0.00bps | 0.00%, 0.00bps | 0.00%, 0.00bps | 0.00%, 0.00bps | 0.00%, 0.00bps |
| Junior tranche: PD, EL | 0.00%, 0.00bps | 0.00%, 0.00bps | 0.00%, 0.00bps | 0.00%, 0.00bps | 0.00%, 0.00bps | 0.00%, 0.00bps |
| Expected IRR (%) | 3.11 | 2.45 | 2.99 | 2.27 | 1.37 | -5.13 |
| Median IRR (%) | 3.13 | 2.46 | 2.99 | 2.28 | 1.39 | -5.08 |
| Standard deviation IRR (%) | 0.36 | 0.38 | 0.37 | 0.38 | 0.40 | 0.69 |
| Pr(complete loss) (%) | 0.00 | 0.00 | 0.00 | 0.00 | 0.00 | 0.00 |
| Pr(IRR < 0) (%) | 0.00 | 0.00 | 0.00 | 0.00 | 0.10 | 100.00 |
| Pr(IRR ≥ 0) (%) | 100.00 | 100.00 | 100.00 | 100.00 | 99.90 | 0.00 |
| Pr(IRR ≥ 2) (%) | 99.90 | 87.00 | 99.40 | 76.30 | 5.80 | 0.00 |
| Pr(IRR ≥ 5) (%) | 0.00 | 0.00 | 0.00 | 0.00 | 0.00 | 0.00 |
| Pr(IRR ≥ 10) (%) | 0.00 | 0.00 | 0.00 | 0.00 | 0.00 | 0.00 |

Figure S11: Incremental cost-effectiveness ratios for various payment models under base-case and pessimistic efficacy assumptions for Zolgensma.

| Base-case efficacy assumptions | Mean treatment costs | Incremental non-treatment health costs* | Total health costs | Incremental QALYs* | Treatment cost/QALY gained | Total cost/QALY gained |
| --- | --- | --- | --- | --- | --- | --- |
| Upfront payment | $1,800,000 | $868,000 | $2,668,000 | 11.77 | $152,931 | $226,678 |
| 5-year PBA |  |  |  |  |  |  |
| 30-year PBA |  |  |  |  |  |  |
| Pessimistic efficacy assumptions | Mean treatment costs | Incremental non-treatment health costs* | Total health costs | Incremental QALYs* | Treatment cost/QALY gained | Total cost/QALY gained |
| Upfront payment | $1,800,000 | $598,000 | $2,398,000 | 6.39 | $281,690 | $375,274 |
| 5-year PBA | $1,681,000 |  | $2,279,000 |  | $263,067 | $356,651 |
| 30-year PBA | $1,269,000 |  | $1,867,000 |  | $198,592 | $292,175 |
| * Source: a model of Zolgensma clinical efficacy and associated costs developed by the Institute for Clinical and Economic Review^1^. | | | | | | |

Criticisms of performance-based annuities

Implicit in the analysis is that long-term PBAs are sufficiently attractive to payers to cause them to seek innovative methods to enable implementation of long-term PBAs. This assumption requires further discussion because PBAs have received extensive criticism in the past. This criticism can be grouped into two main arguments: (1) that measuring the outcomes for risk sharing agreements (RSAs) such as PBAs is administratively burdensome and expensive, and (2) that patient churn makes implementing PBAs challenging. Long-term PBAs also do not allow payers to capture the benefits of competition. This section presents these criticisms and offers rebuttals.

The first main line of criticism against PBAs has been that RSAs, of which the PBA is one example, are costly and difficult to implement^7,8^. These challenges are the result of three distinct problems: high transaction costs, measurement issues, and lack of IT/data infrastructure. The measurement issues are further exacerbated by long-term RSAs because of the need to follow up and monitor patients. One lightening rod of criticism has been the 10-year RSA implemented by the NHS regarding beta-interferon drugs for multiple sclerosis. Critiques have focused extensively on the administrative costs of the scheme, which have been estimated at £7m per year (£1m baseline monitoring costs and £6m to hire MS nurses to monitor outcome)^9^, representing ~15% of total costs per year (£40m/5000 patients for the beta interferon drugs and £7m for administrative costs). The relevance of these criticism to long-term PBAs for durable/curative therapies is questionable. For instance, if nursing costs are assumed to scale with the number of patients monitored, the administrative costs of monitoring 500 type 1 SMA patients in the proposed 30-year PBA is estimated to be $1.93m per year ($1.2m baseline monitoring costs and $0.73m to hire requisite nurses). Compared to the $65m made in annual installment payments, the administrative costs for implementing long-term PBAs are likely small (~3% for these estimates).

Another prominent line of criticism is that PBAs introduce future administrative complications because patients frequently switch insurers^7,8,10^. Critics argue that it is unclear, for instance, whether the old or new insurer would be responsible for continuing annual installment payments on a PBA if the treated patient switches insurers. Further if the new insurer were to be responsible, nothing stops the insurer from backloading payments, thereby shifting the burden to future payers. Such critiques, however, miss the nuance that the patient churn problem is intrinsic to paying for durable/curative therapies. Given the fundamental nature of this problem, efforts are underway to address the patient churn problem in the US^11,12^ and elsewhere. In the meantime, the patient churn problem can be avoided by implementing PBAs in single-payer systems like the English National Health Service. One 2015 survey of 29 payers in the US and Western Europe reported that perceived implementation potential of PBAs in the UK is high^10^.

Long-term PBAs also do not enable payers to realize savings from entry from price reductions that result from market entry of other patented competitor therapies or of generic versions of the initial patented therapy for patients that have already been treated. For chronic therapies, payers realize these cost savings for patients undergoing treatment by switching patients to the cheaper therapy. In contrast, for curative therapies, payers cannot realize these cost savings for treated patients because treated patients do not need another cure for the same disease. Fortunately, competitor/generic risk can be avoided for certain applications of PBAs. In particular, limited competitor/generic risk exists for disease indications for which there is only one durable/curative therapy development. Of the 197 unique indications for which there are gene therapies in human clinical trials, 117 indications (59%) have only one gene therapy in clinical development (Figure S12). While more gene therapies for these 117 indications might be in preclinical development, these figures nonetheless highlight that competitor risk is limited for most gene therapies. The underlying reason for this lack of competitive pressure is that many gene therapies target ultra-orphan diseases (<2/100k prevalence), whose small potential market size likely cannot sustain multiple competitors. However, competitor/generic risk does exist for payers when paying for therapies for non-orphan diseases.

Figure S12: Number of cell/gene therapies in human clinical trials per disease indication.

Data source: PhRMA^13^

| **Disease category** | **Indication** | **#** |
| --- | --- | --- |
| Cancer | multiple myeloma | 18 |
| Cancer | glioblastoma | 16 |
| Cancer | solid tumors | 14 |
| Cancer | AML | 13 |
| Cardiovascular Disease | heart failure | 11 |
| Cancer | ovarian cancer | 10 |
| Cancer | pancreatic cancer | 8 |
| Eye Disorders | retinitis pigmentosa | 8 |
| Cancer | hematologic malignancies | 7 |
| Cancer | lymphoma | 7 |
| Cancer | melanoma | 7 |
| Cancer | prostate cancer | 7 |
| Genetic Disorders | mucopolysaccharidosis I | 7 |
| Blood Disorders | hemophilia A | 6 |
| Cancer | NHL | 6 |
| Cancer | NSCLC | 6 |
| Muscular Dystrophy | Duchenne muscular dystrophy | 6 |
| Transplantation | GVHD | 6 |
| Cancer | head and neck cancer | 5 |
| Cancer | leukemia | 5 |
| Cardiovascular Disease | critical limb ischemia | 5 |
| Diabetes | type 1 diabetes | 5 |
| Eye Disorders | dry age-related macular degeneration | 5 |
| Eye Disorders | wet age-related macular degeneration | 5 |
| Genetic Disorders | mucopolysaccharidosis II | 5 |
| Infectious Diseases | HIV-1 infections | 5 |
| Arthritis/Musculoskeletal Disorders | osteoarthritis | 4 |
| Blood Disorders | beta thalassemia | 4 |
| Blood Disorders | hemophilia B | 4 |
| Cancer | ALL | 4 |
| Cancer | liver cancer | 4 |
| Cancer | DLBCL | 4 |
| Cancer | breast cancer | 4 |
| Cancer | colorectal cancer | 4 |
| Cancer | myelodysplastic syndromes | 4 |
| Cancer | triple negative breast cancer | 3 |
| Cancer | bladder cancer | 3 |
| Cancer | CLL | 3 |
| Cardiovascular Disease | chronic heart failure | 3 |
| Cardiovascular Disease | refractory angina | 3 |
| Eye Disorders | achromatopsia | 3 |
| Eye Disorders | glaucoma | 3 |
| Eye Disorders | X-linked retinitis pigmentosa | 3 |
| Genetic Disorders | glycogen storage disease type I | 3 |
| Infectious Diseases | cytomegalovirus infections | 3 |
| Neurologic Disorders | ALS | 3 |
| Neurologic Disorders | mucopolysaccharidosis III | 3 |
| Neurologic Disorders | traumatic brain injury | 3 |
| Skin | dystrophic epidermolysis bullosa | 3 |
| Transplantation | renal transplant rejection | 3 |
| Alzheimer's Disease | Alzheimer's disease | 2 |
| Arthritis/Musculoskeletal Disorders | intervertebral disc degeneration | 2 |
| Autoimmune Diseases | progressive multiple sclerosis | 2 |
| Autoimmune Diseases | scleroderma | 2 |
| Blood Disorders | Fanconi's anemia | 2 |
| Blood Disorders | severe sickle cell disease | 2 |
| Cancer | EBV-associated cancer | 2 |
| Cancer | Ewing sarcoma | 2 |
| Cancer | nasopharyngeal cancer | 2 |
| Cancer | BPDCN | 2 |
| Cancer | cervical cancer | 2 |
| Cancer | glioma | 2 |
| Cancer | metastatic castration-resistant prostate cancer | 2 |
| Cancer | synovial sarcoma | 2 |
| Cardiovascular Disease | congestive heart failure | 2 |
| Cardiovascular Disease | hypercholesterolemia | 2 |
| Cardiovascular Disease | ischemic heart failure | 2 |
| Cardiovascular Disease | ischemic stroke | 2 |
| Cardiovascular Disease | peripheral artery disease | 2 |
| Crohn's Disease | Crohn's disease | 2 |
| Eye Disorders | choroideremia | 2 |
| Eye Disorders | Stargardt disease | 2 |
| Genetic Disorders | glycogen storage disease type II | 2 |
| Infectious Diseases | hepatitis B | 2 |
| Kidney Diseases | primary hyperoxaluria | 2 |
| Neurologic Disorders | diabetic peripheral neuropathy | 2 |
| Neurologic Disorders | Huntington's disease | 2 |
| Neurologic Disorders | Parkinson's disease | 2 |
| Neurologic Disorders | spinal cord injury | 2 |
| Skin | recessive dystrophic epidermolysis bullosa | 2 |
| Arthritis/Musculoskeletal Disorders | lumbar degenerative disc disease | 1 |
| Arthritis/Musculoskeletal Disorders | muscle injury following arthroplasty for hip fracture | 1 |
| Arthritis/Musculoskeletal Disorders | rheumatoid arthritis | 1 |
| Autoimmune Diseases | complement-mediated diseases | 1 |
| Autoimmune Diseases | inclusion body myositis | 1 |
| Autoimmune Diseases | relapsing remitting multiple sclerosis | 1 |
| Bladder Disorders | overactive bladder | 1 |
| Bladder Disorders | stress urinary incontinence | 1 |
| Bladder Disorders | underactive bladder | 1 |
| Blood Disorders | chemotherapy-induced neutropenia | 1 |
| Blood Disorders | Crigler-Najjar Syndrome | 1 |
| Blood Disorders | neutropenia in AML | 1 |
| Blood Disorders | severe aplastic anemia | 1 |
| Blood Disorders | transfusion-dependent beta thalassemia | 1 |
| Blood Disorders | Wiskott-Aldrich syndrome | 1 |
| Cancer | anal cancer | 1 |
| Cancer | anal HSIL | 1 |
| Cancer | astrocytoma | 1 |
| Cancer | B-cell malignancies | 1 |
| Cancer | B-cell NHL | 1 |
| Cancer | cervical high grade squamous intraepithelial lesion (HSIL) | 1 |
| Cancer | cutaneous T-cell lymphoma (CTCL) | 1 |
| Cancer | gynecologic cancer (combination therapy) | 1 |
| Cancer | indolent NHL | 1 |
| Cancer | liver metastases | 1 |
| Cancer | localized prostate cancer | 1 |
| Cancer | malignant ascites | 1 |
| Cancer | mantle cell lymphoma | 1 |
| Cancer | merkel cell carcinoma | 1 |
| Cancer | metastatic colorectal cancer | 1 |
| Cancer | metastatic liver cancer | 1 |
| Cancer | metastatic solid tumors | 1 |
| Cancer | neuroblastoma | 1 |
| Cancer | peritoneal mesothelioma | 1 |
| Cancer | peritoneal metastases | 1 |
| Cancer | renal cell carcinoma | 1 |
| Cancer | small cell lung cancer (SCLC) | 1 |
| Cancer | squamous cell carcinoma | 1 |
| Cancer | T-cell lymphoma/leukemia | 1 |
| Cancer | urothelial cancer | 1 |
| Cancer | vulvar HSIL | 1 |
| Cardiovascular Disease | acute cardiac ischemia | 1 |
| Cardiovascular Disease | acute myocardial infarction | 1 |
| Cardiovascular Disease | advanced chronic heart failure | 1 |
| Cardiovascular Disease | advanced heart failure | 1 |
| Cardiovascular Disease | cardiovascular disease | 1 |
| Cardiovascular Disease | chronic myocardial ischemia with refractory angina | 1 |
| Cardiovascular Disease | coronary microvascular dysfunction | 1 |
| Cardiovascular Disease | end-stage heart failure | 1 |
| Cardiovascular Disease | hyperlipoproteinemia type Iia (homozygous familial hypercholesterolemia) | 1 |
| Cardiovascular Disease | severe heart damage in congestive heart failure | 1 |
| Eye Disorders | acute primary angle closure glaucoma | 1 |
| Eye Disorders | diabetic macular edema, | 1 |
| Eye Disorders | Leber's congenital amaurosis | 1 |
| Eye Disorders | Leber's hereditary optic atrophy | 1 |
| Eye Disorders | macular telangiectasia type 2 | 1 |
| Eye Disorders | non-arteritic anterior ischemic optic neuropathy | 1 |
| Eye Disorders | ocular hypertension | 1 |
| Eye Disorders | retinitis pigmentosa combined immunodeficiency | 1 |
| Eye Disorders | RLBP1-mutated retinitis pigmentosa | 1 |
| Eye Disorders | X-linked choroideremia | 1 |
| Eye Disorders | X-linked retinoschisis | 1 |
| Genetic Disorders | ADA-SCID | 1 |
| Genetic Disorders | Fabry's disease | 1 |
| Genetic Disorders | mucopolysaccharidosis III A | 1 |
| Genetic Disorders | mucopolysaccharidosis III B | 1 |
| Genetic Disorders | ornithine transcarbamylase deficiency | 1 |
| Genetic Disorders | Usher syndrome 1B | 1 |
| Genetic Disorders | X-linked chronic granulomatous disease | 1 |
| Genetic Disorders | X-linked severe combined immunodeficiency (SCID) | 1 |
| Infectious Diseases | adenovirus infections following hematopoietic stem cell therapy (HSCT) | 1 |
| Infectious Diseases | cytomegalovirus infections (prevention) | 1 |
| Infectious Diseases | cytomegalovirus infections following HSCT (Fast Track) | 1 |
| Infectious Diseases | Ebola infections | 1 |
| Infectious Diseases | viral infections following HSCT (Adv, BK virus, CMV, EBV, HHV6) | 1 |
| Kidney Diseases | acute kidney injury following cardiac surgery | 1 |
| Kidney Diseases | Alport syndrome | 1 |
| Kidney Diseases | autosomal polycystic kidney disease | 1 |
| Kidney Diseases | chronic kidney disease and type 2 diabetes mellitus | 1 |
| Kidney Diseases | diabetic nephropathy in type 2 diabetes | 1 |
| Kidney Diseases | primary hyperoxaluria type 1 | 1 |
| Liver Diseases | acute hepatic porphyria | 1 |
| Liver Diseases | advanced hepatic fibrosis | 1 |
| Liver Diseases | alpha-1 antitrypsin deficiency | 1 |
| Muscular Dystrophy | Becker muscular dystrophy | 1 |
| Muscular Dystrophy | limb girdle muscular dystrophy (type 2B) | 1 |
| Muscular Dystrophy | limb girdle muscular dystrophy (type 2D) | 1 |
| Muscular Dystrophy | limb girdle muscular dystrophy (type 2E) | 1 |
| Neurologic Disorders | aromatic L-amino acid decarboxylase (AADC) deficiency | 1 |
| Neurologic Disorders | cerebral adrenoleukodystrophy | 1 |
| Neurologic Disorders | Charcot-Marie-Tooth disease | 1 |
| Neurologic Disorders | chronic low back pain | 1 |
| Neurologic Disorders | chronic motor deficit due to stroke | 1 |
| Neurologic Disorders | chronic spinal cord injury | 1 |
| Neurologic Disorders | CLN6 neuronal ceroid lipofuscinosis (Batten disease) | 1 |
| Neurologic Disorders | metachromatic leukodystrophy | 1 |
| Neurologic Disorders | polyglutamine spinocerebellar ataxia | 1 |
| Neurologic Disorders | spinal muscular atrophy (SMN1) | 1 |
| Neurologic Disorders | spinal muscular atrophy (SMN2) | 1 |
| Neurologic Disorders | X-linked myotubular myopathy | 1 |
| Other | aging frailty | 1 |
| Other | ATTR amyloidosis | 1 |
| Other | fecal incontinence | 1 |
| Other | immunodeficiency disorders | 1 |
| Other | immunosenescence in aging frailty | 1 |
| Other | radiation-induced xerostomia | 1 |
| Other | unilateral or bilateral severe-to- profound hearing loss | 1 |
| Respiratory | acute respiratory distress syndrome | 1 |
| Respiratory | bronchopulmonary dysplasia (pediatric) | 1 |
| Skin | cutaneous fibrosis | 1 |
| Skin | cutaneous photoaging | 1 |
| Skin | diabetic foot ulcers | 1 |
| Skin | hypertrophic scars | 1 |
| Skin | incisional complications | 1 |
| Transplantation | delayed graft function | 1 |
| Transplantation | in patients with inherited metabolic disorders undergoing HSCT | 1 |
| Transplantation | solid organ transplant support | 1 |

Supplementary endnotes

1. ICER. *Spinraza® and Zolgensma® for Spinal Muscular Atrophy: Effectiveness and Value*.

2. Mendell, J. R. *et al.* Single-Dose Gene-Replacement Therapy for Spinal Muscular Atrophy. *N. Engl. J. Med.* **377**, 1713–1722 (2017).

3. Gregoretti, C. *et al.* Survival of Patients With Spinal Muscular Atrophy Type 1. *Pediatrics* **131**, e1509–e1514 (2013).

4. Zerres, K. *et al.* A collaborative study on the natural history of childhood and juvenile onset proximal spinal muscular atrophy (type II and III SMA): 569 patients. *J. Neurol. Sci.* **146**, 67–72 (1997).

5. United States Mortality Database. Available at: https://usa.mortality.org/. (Accessed: 13th August 2019)

6. Daily Treasury Yield Curve Rates. Available at: https://www.treasury.gov/resource-center/data-chart-center/interest-rates/Pages/TextView.aspx?data=yield. (Accessed: 25th August 2019)

7. Abou-El-Enein, M., Bauer, G. & Reinke, P. The business case for cell and gene therapies. *Nat. Biotechnol.* **32**, 1192–1193 (2014).

8. Danzon, P. M. Affordability Challenges to Value-Based Pricing: Mass Diseases, Orphan Diseases, and Cures. *VALUE Heal.* **21**, 252–257 (2018).

9. Raftery, J. Multiple sclerosis risk sharing scheme: a costly failure. *BMJ* **340**, c1672 (2010).

10. Touchot, N. & Flume, M. The payers’ perspective on gene therapies. *Nat. Biotechnol.* **33**, 902 (2015).

11. MIT FoCUS. *MIT NEWDIGS Research Brief 2018F206- v022-Patient Mobility*. (2018).

12. Butcher, L. Paying for high-cost drugs like you’d pay a mortgage–and with strings attached | Managed Care magazine. *Managed Care Magazine* (2019). Available at: https://www.managedcaremag.com/archives/2019/7/paying-high-cost-drugs-you-d-pay-mortgage-and-strings-attached. (Accessed: 27th August 2019)

13. PhRMA. *Medicines in Development for Cell Therapy and Gene Therapy*. (2018).
